# T Cell Chemo-Vaccination Effects after Repeated Mucosal SHIV Exposures and Oral Pre-Exposure Prophylaxis

**DOI:** 10.1371/journal.pone.0019295

**Published:** 2011-04-26

**Authors:** Ellen N. Kersh, Debra R. Adams, Ae S. Youngpairoj, Wei Luo, Qi Zheng, Mian-er Cong, Wutyi Aung, James Mitchell, Ron Otten, R. Michael Hendry, Walid Heneine, Janet McNicholl, J. Gerardo Garcia-Lerma

**Affiliations:** Division of HIV/AIDS Prevention, Centers for Disease Control and Prevention (CDC), Atlanta, Georgia, United States of America; University of California, San Francisco, United States of America

## Abstract

Pre-exposure prophylaxis (PrEP) with anti-viral drugs is currently in clinical trials for the prevention of HIV infection. Induction of adaptive immune responses to virus exposures during anti-viral drug administration, i.e., a “chemo-vaccination” effect, could contribute to PrEP efficacy. To study possible chemo-vaccination, we monitored humoral and cellular immune responses in nine rhesus macaques undergoing up to 14 weekly, low-dose SHIV_SF162P3_ rectal exposures. Six macaques concurrently received PrEP with intermittent, oral Truvada; three were no-PrEP controls. PrEP protected 4 macaques from infection. Two of the four showed evidence of chemo-vaccination, because they developed anti-SHIV CD4^+^ and CD8^+^ T cells; SHIV-specific antibodies were not detected. Control macaques showed no anti-SHIV immune responses before infection. Chemo-vaccination-induced T cell responses were robust (up to 3,940 SFU/10^6^ PBMCs), predominantly central memory cells, short-lived (≤22 weeks), and appeared intermittently and with changing specificities. The two chemo-vaccinated macaques were virus-challenged again after 28 weeks of rest, after T cell responses had waned. One macaque was not protected from infection. The other macaque concurrently received additional PrEP. It remained uninfected and T cell responses were boosted during the additional virus exposures. In summary, we document and characterize PrEP-induced T cell chemo-vaccination. Although not protective after subsiding in one macaque, chemo-vaccination-induced T cells warrant more comprehensive analysis during peak responses for their ability to prevent or to control infections after additional exposures. Our findings highlight the importance of monitoring these responses in clinical PrEP trials and suggest that a combination of vaccines and PrEP potentially might enhance efficacy.

## Introduction

Clinical trials are currently underway or are being completed to evaluate the efficacy of pre-exposure prophylaxis (PrEP) with anti-retroviral (ARV) drugs for the prevention of HIV infection [1], [2], [3], [4]. It is possible that HIV exposure during prophylaxis can stimulate the immune system and induce adaptive immunity in the absence of productive infection. This would be akin to the observation that HIV-exposed, uninfected (EU) individuals can harbor HIV-specific immune responses associated with protection from infection ([5], and reviewed by Kulkarni et al. [6]). The effect could be viewed as “chemo-vaccination”, with natural HIV exposures providing antigenic stimulation to the immune system, while chemicals (ARVs) prevent or limit viral replication and productive infection. A chemo-vaccination effect could be an added benefit that contributes to the overall efficacy of PrEP, even in individuals with low adherence to antiviral drug regimens. Benefits of chemo-vaccination could include prevention of virus acquisition, but also improved viral control should breakthrough infections occur. Alternatively, immune activation due to virus exposure during PrEP could raise susceptibility to HIV infection by recruiting activated, HIV-specific CD4^+^ target cells to mucosal surfaces, thus potentially facilitating infection during subsequent exposures [7], [8] or increasing replication after virus transmission [7], [9]. It is therefore essential to understand if chemo-vaccination can occur during PrEP and how this can modulate susceptibility to infection. Non-human primate models of mucosal SIV or SHIV exposure are ideal models to assess all these questions under highly controlled conditions. The type and duration of immune responses induced by PrEP during repeated mucosal virus exposures can also be dissected in these models.

We have previously used rhesus macaques to closely model human sexual HIV exposures [10] by repeatedly exposing macaques at vaginal or rectal mucosal surfaces with low doses of a CCR5-using SHIV strain. We subsequently employed this repeat-low-dose (RLD) model to extensively test efficacy of various ARV drugs in different PrEP schedules and doses under conditions relevant to humans [11], [12], [13]. We now use the RLD model to study adaptive immune responses induced by an intermittent PrEP regimen with Truvada, a combination of the HIV-1 reverse transcriptase inhibitors emtricitabine (FTC) and tenofovir disoproxyl fumarate (TDF) [14]. The PrEP regimen was chosen to model relevant scenarios for human clinical trials, rather than to design ideal conditions for the priming of immune responses. We find that SHIV-specific T cells, but not antibodies, appear following virus exposures during PrEP. This confirmed earlier T cell chemo-vaccination reports in nonhuman primates receiving topical ARVs by others and us [13], [15]. We expand these earlier observations by now clearly documenting the absence of responses before virus challenge and by establishing a cut-off for positive responses elicited by chemo-vaccination based on pre-exposure baseline responses. Furthermore, we now dissect the time course of anti-SHIV T cell appearance and disappearance, their epitope specificity, CD4/CD8 composition, and ability to produce multiple cytokines. Moreover, using a macaque model allowed us to re-challenge a subset of the macaques after completion of PrEP to directly test for protective effects of immune responses.

## Results

### Repeated mucosal virus exposures and intermittent, oral PrEP

**Figure 1:**
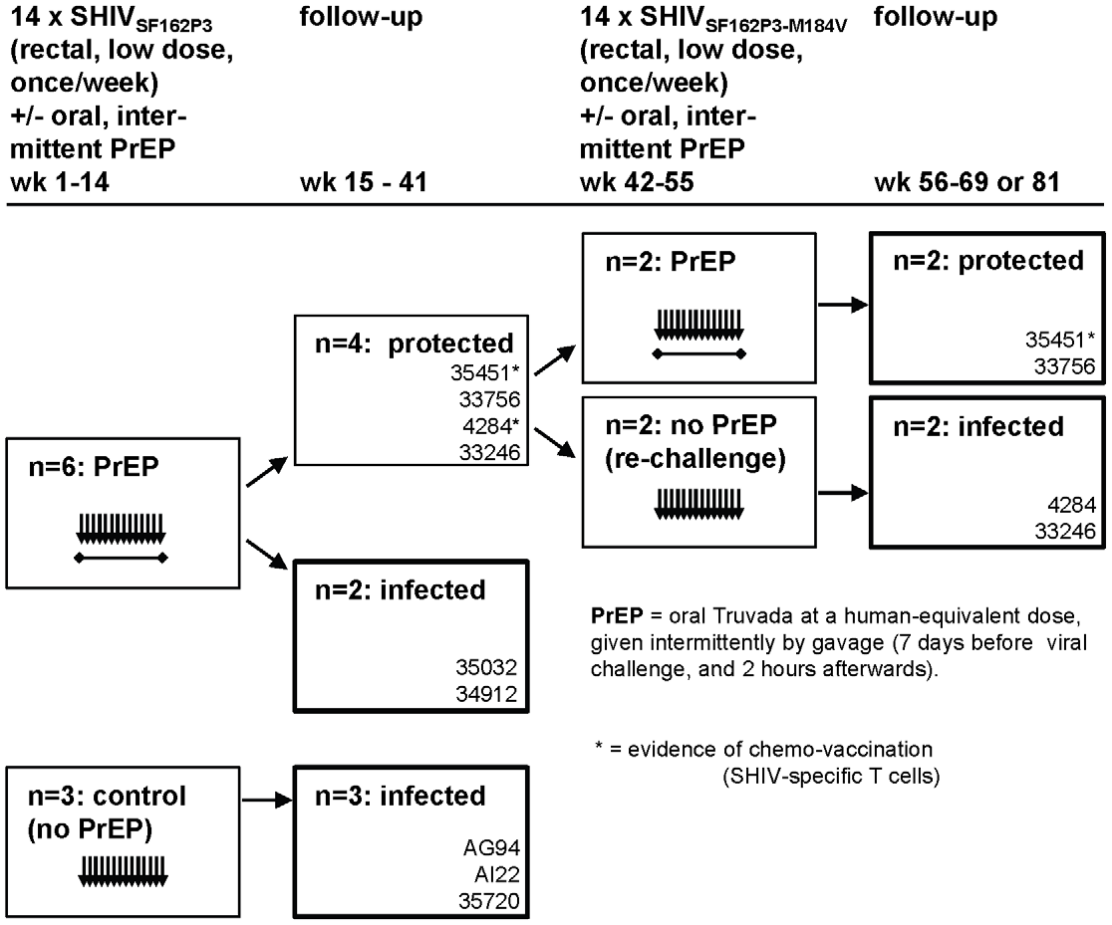
Experimental Design. SHIV-specific T cells were measured during the indicated experimental procedures. Arrows indicate repeated viral exposures, horizontal lines depict intermittent, oral PrEP. PrEP consisted of human-equivalent doses of oral Truvada. Each virus exposure was flanked by a waning drug dose of 7 days prior, and one drug dose administered 2 hours after exposure, as a model for intermittent PrEP use in humans. Bolded rectangles highlight final outcomes of SHIV challenges. Numbers in lower right corners refer to macaque identifications (IDs).

We sought to determine whether a relevant intermittent PrEP regimen designed for human application induces T cell immunity. Figure 1 outlines the study protocol used to model human sexual transmission of HIV during PrEP, and indicates outcome of viral exposures. We used the RLD rectal SHIV-exposure macaque model and a partially effective PrEP regimen of intermittent, oral Truvada administration [16]. Four PrEP-treated macaques remained uninfected, while two were infected after 4 and 14 rectal SHIV exposures, respectively. The three untreated control macaques became infected after 1, 3, or 5 exposures, consistent with previous findings in 29 additional controls receiving the same virus-only rectal treatment [16]. Compared to a total of 32 untreated controls, the PrEP regimen reduced the risk of infection by 9.3-fold (p = 0.003) [16].

### Enumeration of SHIV-specific T cells elicited by chemo-vaccination during and after repeated SHIV exposures

**Figure 2:**
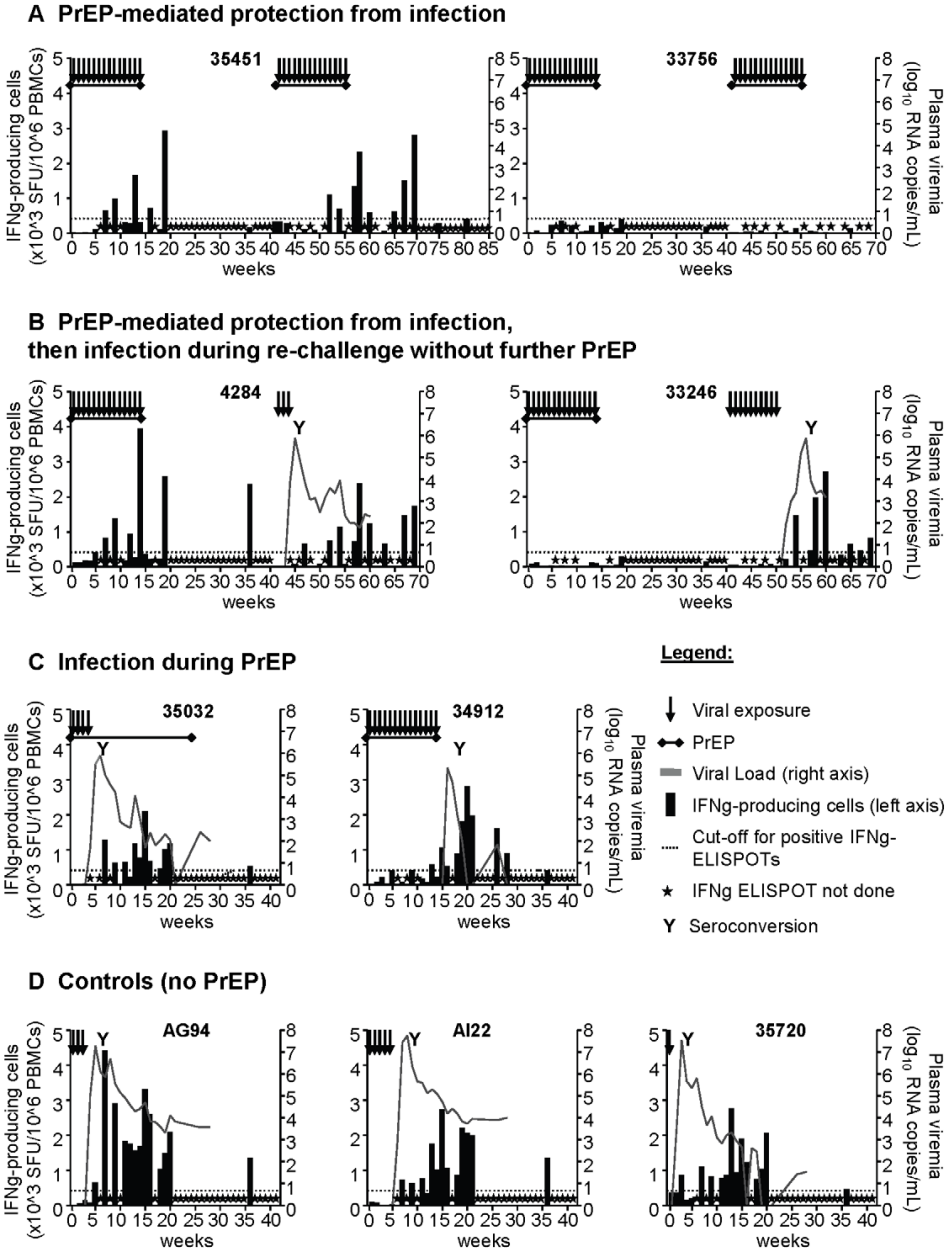
Chemo-vaccination effect. SHIV-specific T cells are induced in two PrEP-protected macaques during PrEP and virus exposures. PrEP protected four macaques from infection during 14 SHIV exposures in weeks 1–14 (**A** and **B**), while two became infected despite PrEP (**C**); three macaques were controls (**D**). SHIV-specific T cells were determined by IFNγ-ELISPOT. The black bars represent the number of specific T cells as a sum of responses to 14 SHIV-derived peptide pools for antigenic simulation, measured in SFU (spot forming units, left axis). Grey lines depict plasma viremia (right axes). The graphs represent data from individual macaques, their identification codes are bolded. The dotted lines are cut-off values for positive T cell responses. Two PrEP-protected macaques (35451 (**A**), 4284 (**B**)) showed signs of chemo-vaccination. During re-challenge with 14 SHIV exposures in weeks 42–55, additional PrEP protected macaques 35451 and 33756 (**A**). Chemo-vaccinated macaques 4284 and 33246 were not protected from SHIV infection (**B**). Throughout the study, anti-SHIV antibodies were determined every 6 weeks (weeks 1–41) or 4 weeks (weeks 42–69). “Y” indicates time of seroconversion.

During 14 initial virus exposures and 27 weeks of follow-up, we enumerated SHIV-specific T cells in peripheral blood by IFNγ-ELISPOT, using 14 SHIV-derived peptide pools to stimulate fresh PBMCs. Before antigen exposure, in study week 0, the combined T cell response directed at these antigen pools ranged from 0 (animals 35451, 33756, 35032) to 355 SFU/10^6^ PBMCs (animal 35720) (Fig. 2A–D), with a mean 73 SFU/10^6^ PMBCs. To discriminate positive T cell responses from background, we used a cut-off value of 417 SFU/10^6^ PMBCs, equal to all animals' mean value in week 0 plus 3 standard deviations. T cell responses above this cut-off in the absence of productive SHIV infection were defined as evidence for chemo-vaccination. Two of the four PrEP-protected macaques developed T cell responses during the 14 weeks of virus challenges (35451 and 4284) (Fig. 2A, B), and therefore showed evidence of chemo-vaccination. The first responses in macaques 4284 and 35451 were observed during week 7, after the macaques had already received 6 virus exposures. The responses increased over time, reaching a peak 3940 SFU/10^6^ PBMCs (4284) in week 14, or 2930 SFU/10^6^ PBMCs (35451) in week 19. Responses were only observed intermittently, e.g. they were detectable in weeks 7, 9, 12, and 14, but not 11 and 13 in macaque 4284, and similarly detectable in weeks 7, 9, 13, but not 11, 12, and 14 in macaque 35451.

To better understand the longevity of the T cell responses seen in the two chemo-vaccinated macaques, we continued to monitor IFNγ-producing cells intermittently for up to study week 41, 27 weeks after the last virus challenges. Figure 2B shows that macaque 4284 still had SHIV-specific T cells at weeks 19 and 36, but not at week 41. Macaque 35451's T cell responses were only examined at weeks 19 and 36, and responses were only detectable at week 19 (Fig. 2A). Thus, the minimal documented T cell longevity was 22 and 5 weeks after last antigen exposure, respectively. Due to the low assay frequency, it is possible that T cells persisted longer, but they had definitively subsided within 27 and 22 weeks of last antigen exposure in both macaques.

Two macaques, 35032 and 34912, became infected after 3 or 14 virus exposures, respectively, despite PrEP (Fig. 2C). Macaque 35032 showed no T cell responses during exposures, but developed them after infection as expected. In contrast, macaque 34912 had one response at week 13 before SHIV infection, and thus displayed chemo-vaccination effects according to our definition. The response was weak (585 SFU/10^6^ PBMCs). Thus, taken together, three of the six PrEP-treated macaques had chemo-vaccination-induced T cells at least once during the trial. The T cell response of 34912 was present three weeks before viral RNA was first detected in week 16, indicating that the weak T cell response did not prevent infection. Control macaques had no SHIV-specific T responses prior to their infection after 1, 3, or 5 exposures, as previously observed in an exposed, uninfected macaque, and in several macaques infected late during 14 repeated rectal exposures [17]. After infection, control macaques developed T cell responses as expected (Fig. 2D). Responses peaked at 4405, 2725, and 2770 SFU/10^6^ PBMCs at weeks 7, 15, 13, respectively, with absolute numbers that were similar to the chemo-vaccination levels of the exposed but uninfected macaques.

In contrast to T cell immunity, SHIV-specific binding antibodies appeared only as a result of SHIV infection, but not following chemo-vaccination in the absence of infection. Seroconversion happened within up to 4 weeks of infection and the development of T cell responses (as indicated by the letter “Y” in Figure 2). PrEP-protected, chemo-vaccinated macaques did not have anti-SHIV antibodies when analyzed in weeks 12, 14, 20, and 40 (data not shown).

### Rapidly changing epitope specificity of T cells elicited by chemo-vaccination during and after SHIV exposures

**Figure 3:**
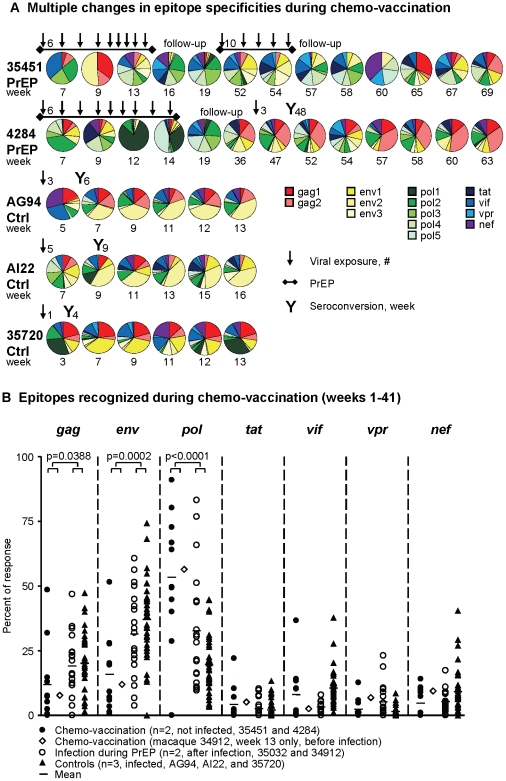
Epitope specificity of T cells induced by chemo-vaccination. **A**: T cell specificities in PrEP-protected macaques 4284 and 35451 change rapidly during SHIV exposures and PrEP, while they remain more consistent in infected control macaque AG94, and in 4284 after infection. T cell specificities were determined by IFNγ-ELISPOT with 14 peptide pools represented by the indicated colors. The pie charts depict percentages of contributions to the T cell response. Arrows indicate time point of virus exposures, adjacent numbers indicate how many exposures were given. Seroconversion is recorded by “Y”; numbers indicates the study week of seroconversion. **B**: T cells induced by chemo-vaccination appear focused on epitopes derived from the *pol* region. IFNγ-ELISPOT responses to 14 peptide pools were combined for the indicated gene products; their contribution to the response (all 14 peptide pools) was calculated. All IFNγ-ELISPOT results from week 1–41 are depicted. N refers to number of macaques in the 4 specified groups. P-values were obtained by unpaired, two-sided student's t-tests comparing all results before infection (12 time-points from 3 chemo-vaccinated macaques, filled circles and open diamonds combined) to those after infection (56 time-points from 5 macaques in control or PrEP-infected groups, open circles and filled triangles combined).

The relative contribution of each of the 14 peptide pools to the overall T cell response changed in chemo-vaccinated macaques 4284 and 35451 during virus exposures and concurrent PrEP (Fig. 3A). For example, in macaque 4284, the most dominantly recognized peptide pools changed from pol 2→pol 4→pol 1→pol 5 in weeks 7, 9, 12, and 14, respectively. In contrast, specificities in infected control macaques remained more stable during a similar time frame (Fig. 3A, macaques AG94, AI22, 35720). For example, in control macaque AG94, T cells underwent one early shift in focus from the nef to the env 2 peptide pool, but then remained focused on env 2 throughout the study.

We addressed whether chemo-vaccination-induced T cells had different epitope specificities than T cells induced during SHIV infection. In chemo-vaccinated, PrEP-protected macaques, a mean 54% of activated T cells were directed towards pol sequences during 41 weeks of virus exposures and follow-up, compared to only 24% in macaques infected during PrEP or as controls (p<0.0001, Fig. 3B). A focus on pol was also observed on the one occasion of T cell responses in PrEP-treated macaque 34912 before its infection, as is indicated in Fig. 3B. In comparison, only 28% of 34912's T cells recognized pol epitopes on the last time point of T cell analysis (study week 28) after SHIV infection (Fig. 3B, and data not shown). Only 12% and 16% of the T cells in chemo-vaccinated macaques focused on gag and env, respectively, while 20% and 35% were focused on gag and env in infected PrEP-treated or control macaques (p = 0.0388 and p = 0.0002 for gag and env, respectively). Thus, chemo-vaccination-induced anti-SHIV T cells indeed had a shifted epitope focus compared to productive SHIV infection.

### Cytokine production by CD4^+^ and CD8^+^ T cells associated with chemo-vaccination during and after SHIV exposures

**Figure 4:**
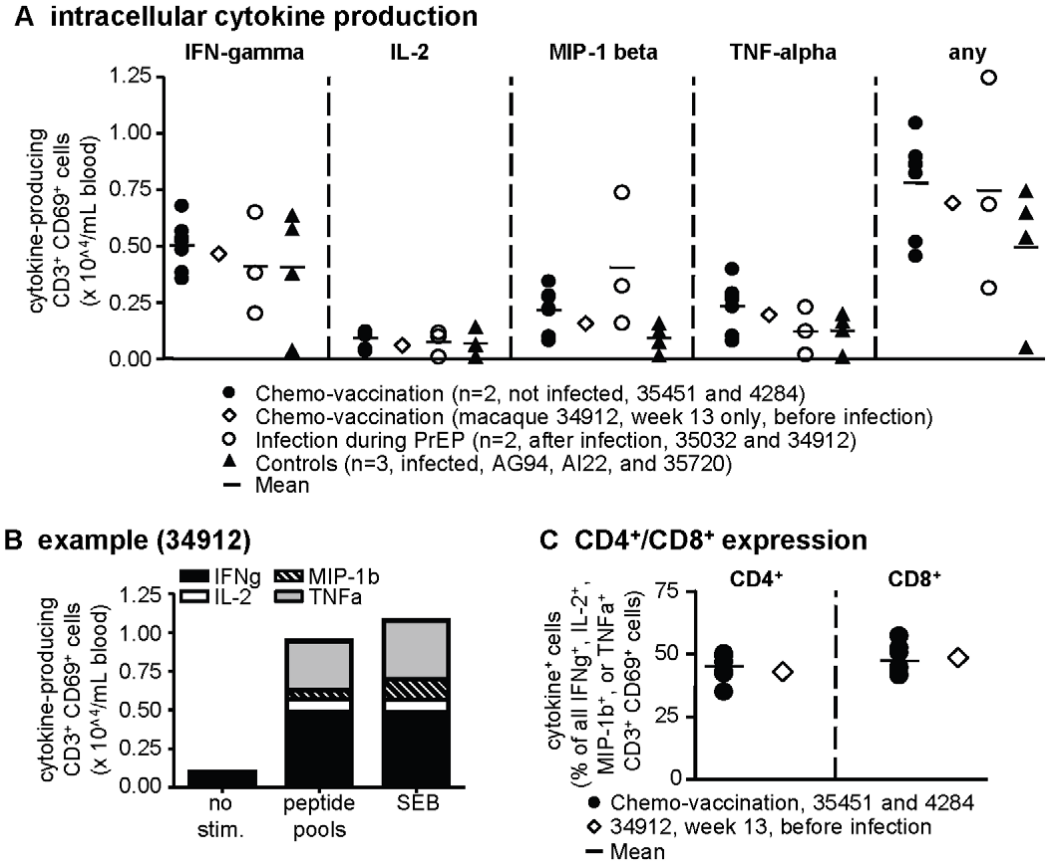
Cytokine production of CD4^+^ and CD8^+^ T cells induced by chemo-vaccination. Intracellular production of IFNγ, IL-2, MIP-1β, or TNFα was measured by flow cytometry after in-vitro incubation of freeze-thawed cells with the two dominant peptide pools as determined by previous IFNγ-ELISPOT. We gated on CD3^+^ and CD69^+^ (**A, B**), or on CD3^+^, CD69^+^, and CD4^+^ or CD8^+^ (**C**), and determined the number of cells with intracellular production of any of the factors, regardless of whether they simultaneously produced the remaining 3 factors. “Any” refers to cells producing any of the indicated factors, not necessarily all simultaneously. Samples from infected controls or infected PrEP-treated macaques were from peak viremia or 6 weeks thereafter, whenever available. Such samples are not shown for CD4/CD8 analysis, because CD4^+^ cells significantly decline depending on the stage of SHIV infection. (**B**) Representative example of results obtained with cells from macaque 34912 before its infection, without stimulation (“no stim.”), with the two dominant peptide pools, or with SEB for polyclonal stimulation.

To analyze functional properties of PrEP-induced T cells, we tested their ability to produce intracellular IL-2, MIP-1β, TNFα, and IFNγ. The two dominantly recognized peptide pools at each time point were determined by ELISPOT, and were then used to optimally stimulate T cells. Figure 4A depicts the number of T cells with intracellular induction of IL-2, MIP-1β, TNFα, or IFNγ from all of the macaques (chemo-vaccinated/uninfected, or infected during PrEP or as controls). Chemo-vaccination induced cytokine-producing T cells in similar numbers as SHIV infection of controls or PrEP-breakthrough macaques, indicating that chemo-vaccination-induced T cells are functional with regards to cytokine production. Macaque 34912's T cells showed no selective inability to produce cytokines before it became infected, indicating that the lack of protection from subsequent infection was not due to inability to produce cytokines. In Figure 4B, a representative graph displays the induction of cytokine-production by peptide or superantigen-stimulation in cells from 34912. Antigen stimulation resulted in substantial increases in intracellular production of all the four factors. A fraction of T cells were also able to simultaneously produce cytokines (data not shown, and see below). SHIV-specific, cytokine-producing T cells in blood consisted of both CD4^+^ and CD8^+^ T cells in chemo-vaccinated macaques 4284, 35451, and in 34912 before infection (Fig. 4C). Macaque 34912 had no unusual expansion of CD4^+^ blood cells which could have provided a larger pool of infection target cells (Fig. 4C), suggesting that a greater availability of susceptible CD4^+^ cells was not the cause of subsequent infection.

### Re-challenge of previously PrEP-protected macaques with SHIV; enumeration of anti-SHIV T cells and their epitope specificities

After 41 weeks of study, when SHIV-specific T cell activities had subsided to undetectable levels in blood, the four PrEP-protected, uninfected macaques were enrolled into another PrEP trial that evaluated the impact of drug resistance on PrEP efficacy [14] (Fig. 1). In the subsequent trial, the animals received a second round of SHIV exposures 28 weeks after they were last exposed in the previous study, and we examined whether a history of SHIV exposures, PrEP, and/or T cell responses altered the outcome of additional SHIV challenges.

Two of the macaques (4284 and 33246) were enrolled into a control arm receiving no further PrEP during 14 additional RLD challenges using SHIV_SF162P3-M184V_. The other two macaques (35451 and 33756) were virus challenged while receiving PrEP with Truvada. Each of the study arms included one macaque with and one without a history of previous SHIV-specific T cells (Fig. 1, 2).

Both control macaques became infected (Fig. 2B). These 2 macaques (4284, 33246) were identical at 8 Mamu alleles known to impact SIV pathogenesis (see Materials and Methods). Macaque 4284, with a history of T cell responses up to week 36, but no responses thereafter, became infected after 3 virus exposures. Macaque 33246 without any previous SHIV-directed T cells was infected after 10 exposures. Infections after 3 and 10 exposures are within the normal range of exposure-naïve animal infections with this virus strain [14]. The history of SHIV-specific T cells in this animal, therefore, did not protect from or accelerate virus acquisition at this time-point, when T cell responses had subsided. The two macaques had remarkably similar levels of viremia (Fig. 2B), which peaked at 5.0 or 5.5×10∧6 viral copies/mL in blood in 4284 and 33246, respectively.

Neither of the two animals in the PrEP/SHIV exposures arm became infected (Fig. 2A). The virus exposures during PrEP again induced SHIV-specific T cell responses in chemo-vaccinated 35451, but still none in macaque 33756 (Fig. 2A). Thus, macaque 33756 received 28 SHIV exposures and remained uninfected, but did not exhibit any SHIV-specific blood T cells in 25 independent assays. In macaque 35451, which similarly received 28 SHIV exposures and remained uninfected, T cell responses were not easily re-called, and only became detectable in blood after 10 additional virus exposures.

T cells were detected intermittently during virus exposures, as observed after the first 14 SHIV exposures. In macaque 35451, anti-SHIV responses were detected at weeks 52 and 54, but not 55, followed by consistent detection in the follow-up phase (weeks 57–69). T cell epitope focus continued to shift between detection times (Fig. 3A).

### T cell differentiation parameters in chemo-vaccinated macaque 35451

**Figure 5:**
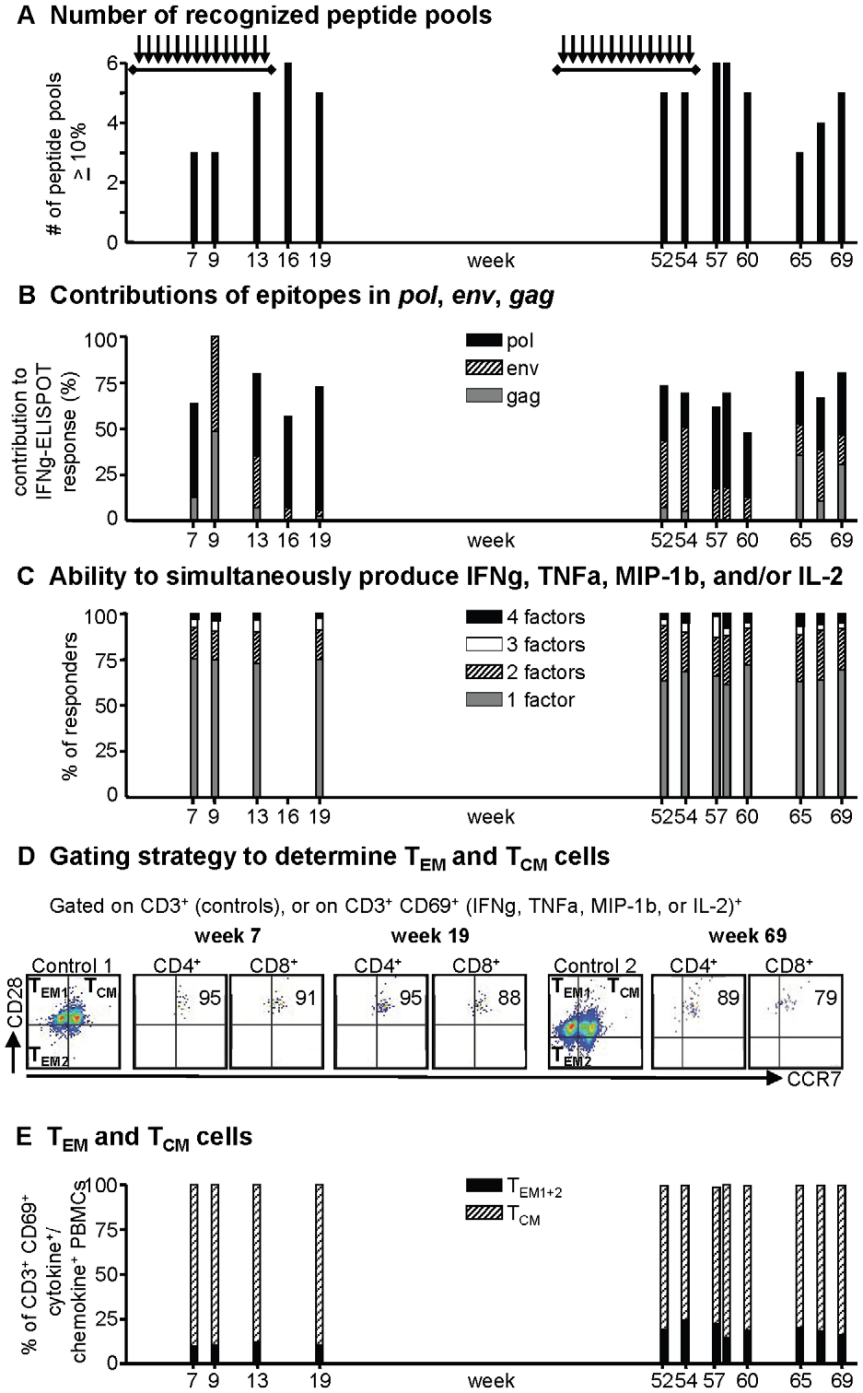
Epitope specificity and differentiation parameters of anti-SHIV T cells in chemo-vaccinated macaque 35451. **A: Number of recognized peptide pools:** As a measure of the breadth of T cell diversity, the number of peptide pools recognized was examined. Only pools recognized with 10% or more of the anti-SHIV T cell ELISPOT response are shown. **B: Contributions of epitopes in** ***pol*** **,** ***env*** **,** ***gag*** **:** T cells to products of *pol*, *env*, and *gag* are depicted as percentage of the total IFNγ-ELISPOT response. **C. Ability to simultaneously produce multiple cytokines/chemokines.** Intracellular production of IFNγ, TNFα, MIP-1β, and/or IL-2 (“factors”) was measured by flow cytometry after gating on CD3^+^ CD69^+^ cells. Freeze-thawed PBMCs were cultured with 2 dominant peptide pools as determined by ELISPOT for each time point. We determined the number of cells that produced any of the factors (“responders”), and then analyzed whether the cells made any one factor alone, any two or three factors, or all four factors simultaneously. **D. Gating strategy for determination of anti-SHIV effector and central memory T cells** (T_EM_ and T_CM_, respectively). Cells were stimulated as described in **C**. For the indicated control analyses, CD3^+^ gated cells were analyzed for CD28 or CCR7 expression, thus defining transitional T_EM1_, T_EM2_, and T_CM_ quadrants for samples analyzed on the same day, i.e. from weeks 7 or 19 (control 1) or from week 69 (control 2). The same quadrants were applied to anti-SHIV T cells (CD3^+^, CD69^+^, and [IFNγ^+^, TNFα^+^, MIP-1β^+^, and/or IL-2]^+^), after further gating on CD4^+^ or CD8^+^ cells. The numbers in the upper right quadrants refer to T_CM_ cells as percentage of anti-SHIV CD4^+^ or CD8^+^ cells. **E: T_EM_ (T_EM1_ and T_EM2_) and T_CM_ cells are shown as percentage of anti-SHIV T cells (CD4^+^ or CD8^+^ cells)**, as determined by flow cytometry described in **C** and **D**. Arrows indicate virus exposures, horizontal bars depict PrEP treatment.

Macaque 35451 was the most extensively chemo-vaccinated macaque in our study. We further dissected epitope specificity and differentiation parameters in this macaque (Fig. 5). The number of peptide pools recognized over the entire study is shown in Fig. 5A. Epitope diversity appeared similar during and following re-challenge (challenges 15–28), because an average 4.9 peptide pools were recognized with more than 10% of the total T cells during weeks 52–69, compared to an average 4.4 pools in weeks 7–19. The relative contribution of *pol*-, *env*-, and *gag*-specific T cells in 35451 over the 69 week study is shown in Fig. 5B. T cells in this uninfected macaque remained largely focused on epitopes in *pol* gene products, but the average contribution to the T cell response was now only 33% in weeks 52–69, compared to 42% in weeks 7–19. To further study T cell differentiation, we analyzed whether simultaneous production of multiple cytokines increased, as happens during functional T cell maturation. We determined the percentage of SHIV-specific T cells producing all four measured factors simultaneously, or any 3 or 2 of them simultaneously (Fig. 5C). The percentage of anti-SHIV T cells producing all four measured factors (IFNγ, TNFα, MIP-1β, and IL-2) simultaneously increased slightly from an average 3% in weeks 7–19 to 5% in weeks 52–69, while cells producing any of these factors alone decreased slightly from 75 to 66%. T cell differentiation can also be examined by analyzing contributions of cytokine-producing effector memory (T_EM_ = CD3^+^ CD69^+^ [IFNγ, TNFα, MIP-1β, and/or IL-2]^+^, CD28^high/low^, CCR7^−^) and central memory (T_CM_ = CD3^+^ CD69^+^ [IFNγ, TNFα, MIP-1β, and/or IL-2]^+^, CD28^high^, CCR7^+^) cells using flow cytometric methods [18]. Anti-SHIV CD4^+^ and CD8^+^ T cells were predominantly T_CM_ cells at all time points following chemo-vaccination in macaque 35451 (Fig. 5D shows flow cytometry data for select time points, and Fig. 5E displays results for all time points), and also in macaque 4284 (data not shown). The contribution of T_EM_ (transitional T_EM1_ and fully differentiated T_EM2_ populations combined) to anti-SHIV CD4^+^ or CD8^+^ T cells rose slightly from 11% to 19% in weeks 7–19 compared to weeks 52–69, respectively, in macaque 35451.

Anti-SHIV T cells were still detectable in week 69 in macaque 35451, 14 weeks after last virus challenge, but no longer in study weeks 75 and 81 (Figure 2). Thus, longevity was again limited, and declined to undetectable levels within 20 weeks after last virus challenge.

## Discussion

We show that PrEP with anti-retrovirals can facilitate the development of adaptive T cell immunity in response to mucosal virus exposures. This “chemo-vaccination” effect was documented in detail by analyzing incidence, duration, epitope specificities, functional properties, differentiation markers, and protective effects of T cells. This confirms earlier chemo-vaccination reports either during pre-exposure prophylaxis [15], [13], or following effective anti-retroviral treatment begun shortly after inoculation [19]. We significantly expanded on earlier studies by using a cut-off for positive anti-viral T cell responses based on background measurements in naïve animals. This allowed us to demonstrate that chemo-vaccination-induced T cell responses are not merely pre-existing background responses with cross-reactivity to SHIV-derived antigens.

Chemo-vaccination induced anti-SHIV T cells in two of four PrEP-protected macaques, and in one of two macaques before they failed PrEP. One reason for the incomplete response rates could be variations in viral antigen presentation in individual macaques, e.g., due to MHC allelic variability. It is noteworthy that macaque 35451 with strong T cell responses was the only study macaque harboring Mamu-B08, an allele associated with superior control of SIV [20]. The other macaque with chemo-vaccination-induced T cells (4284), on the other hand, was not unique in its genetic MHC make-up. It is possible that T cell responses developed, but were not detected in all macaques, due to different induction kinetics, trafficking behavior, or compartmentalization.

A major limitation was the small number of animals at the beginning of the study, and further subdivision of animals due to different experimental outcomes. Only one animal with anti-SHIV T cells could be re-exposed to virus, and only when its T cell responses had subsided. Nevertheless, the in-depth description of chemo-vaccination and long follow-up time in this study provide useful information for future study designs to further examine incidence and consequences of chemo-vaccination in macaques and in limited samples from human clinical PrEP trials. In future studies, it would also be worthwhile to comprehensively evaluate mucosal specimens, and tissue- or lymphatic tissue-resident lymphocytes.

We characterized chemo-vaccination induced T cells. Life-span of the cells was limited to less than 27 weeks, while memory T cells induced by potent vaccines or infection can last for years if not a lifetime [21]. Re-stimulation of previously expanded T cells appeared impaired, because dominant epitope specificities changed rapidly. This suggests that immune memory qualities were not well developed. Memory T cells were mostly of T_CM_ phenotype, while superior protection from and control of SIV by mucosal T_EM_ cells has been reported [18]. Lastly, we noticed a focus on *pol* epitopes, rather than *env* and *gag*. Anti-*pol* immune-reactivity can be effective [22], but efficient viral control is better documented for *gag* –directed T cells [23]. It is noteworthy that a study in HIV^+^ individuals exposed to other HIV strains by their HIV^+^ partners, but not super-infected, also found T cells focused on *pol* products [24]. In addition, dominant T cell responses to *pol* rather than *env* have been reported for EU individuals [25]. Focus on *pol* might therefore be a feature of T cells elicited by priming in the absence of productive infection. It is possible that different tests for T cell differentiation and function (e.g., for proliferative or cytotoxic capabilities) would have revealed additional T cell characteristics, but no additional specimens were available for such analyses.

The high frequency of virus exposures likely prevented efficient functional T cell memory maturation and survival. It is important to emphasize, however, that the study was not designed to optimally induce T cell immunity. Rather, we addressed what kind of immunity is induced by physiologic virus exposures concurrent with partially effective PrEP. Thus, we consider our results relevant for the type of T cell immunity that can be expected following an intermittent PrEP regimen in sexually active people.

Having a history of SHIV-specific T cells due to chemo-vaccination did not protect macaque 4284 from SHIV infection, nor did it delay or significantly accelerate its infection. Ensuing viremia was remarkably similar in another re-challenged macaque (33246), although 33246 had never shown anti-SHIV T cell responses. Both macaques were identical at eight MHC alleles known to impact viral control. Limitations of these studies include: Only one animal was tested, and re-exposure occurred when peripheral blood virus-specific T cells had waned. Another macaque, 34912, became infected after 14 viral exposures, despite weak T cell responses after the 13^th^ exposure. It is unclear whether the animal was already in the viral eclipse phase when T cells were detected. Further research is needed to determine a potentially increased or decreased infection risk after chemo-vaccination during subsequent virus exposures.

T cells matured further in macaque 35451 after resting and undergoing more chemo-vaccination, but it is not clear whether minor changes in epitope specificities and memory phenotype significantly affected anti-viral functions. Chemo-vaccination-induced T cell immunity could possibly be improved by combining PrEP with a vaccine targeted to induce specific immune pathways. A complex vaccine with an antibody-inducing component could complement the observed lack of antibodies; another component could accelerate limited T_EM_ differentiation. Deliberately skewing T cells towards T_EM_ phenotype would most likely also increase mucosal anti-viral responses [18], [21]. Moreover, the RV144 HIV vaccine trial suggested that vaccine efficacy may have decreased over the first year after vaccination [26]. If a vaccine is administered prior to PrEP, chemo-vaccination could possibly boost and extend limited vaccine efficacy. Thus, combination of these two HIV prevention methods could have additive or synergistic effects and raise their combined efficacy significantly. These issues could be addressed by further research in nonhuman primate models employing comparative groups of single or combined prevention modalities, administered during controlled virus exposures.

Anti-SHIV T cells were composed of CD4^+^ and CD8^+^ cells, suggesting that antigen presentation likely occurred in MHC class I and II pathways. It is possible that antigen presentation was mostly the result of uptake of virus particles by resident antigen presenting cells through endocytic mechanisms. Many experimental SHIV stocks contain a large proportion of replication-defective, non-infectious, or severely attenuated virions; these may induce immune responses by this mechanism. We previously found no anti-SHIV T cell responses in macaques exposed rectally up to 14 times to virus without concurrent PrEP [17], although they have been observed after vaginal exposure [27]. It is possible that PrEP-treated macaques experienced a low level of initial virus replication that remained local, and did not result in systemic infection due to the anti-retroviral action of Truvada. Because the PrEP regimen was partially effective, and did not block infection in all macaques, there may have been more transient/abortive infection than during highly effective PrEP, resulting in stronger induction of immune responses, as has been documented in earlier PrEP studies of perinatal SIV infection by Van Rompay et al [28]. Even if initial local replication occurred, it likely did not persist in a suppressed state, because we did not detect it using three assays (detection of plasma viral RNA load, proviral DNA in PBMCs, and serum anti-SHIV antibodies) during extended follow-up (data not shown). In addition, we previously reported no evidence for occult infection in similarly PrEP-protected macaques after in-vivo depletion of CD8^+^ T cells [29].

It is not clear why chemo-vaccination induced no B cell immunity despite considerable T cell immunity. B cell induction requires CD4^+^ T cell help. High-avidity memory CD4^+^ T cells are induced by more prolonged antigenic stimulation than CD8^+^ cells [30]. It is possible that chemo-vaccination-induced anti-SHIV CD4^+^ T cells did not differentiate enough to become efficient B cell helpers. Alternatively, viral antigens possibly did not reach lymphoid tissues necessary for B cell maturation [31]. Mucosal secretions were not tested for the presence of anti-viral antibodies, and it is therefore possible that mucosal but not systemic antibodies were induced by chemo-vaccination. In addition, low-level SHIV-specific antibodies may have been below the limit of detection of the Bio-Rad 1/2 plus O EIA assay, although this assay is considered more sensitive than commercial Western Blotting techniques for the diagnosis of sero-conversion.

It is currently unclear whether human iPrEx [4] or other PrEP trial participants experienced chemo-vaccination effects and whether this is impacted by HIV exposure frequency. Exposure interval effects have been observed in EU commercial sex workers, including a group who lost T cell responses after taking a 2-month or longer break from sex work [5]. Potential protection associated with chemo-vaccination could be determined when individuals are retrospectively grouped into HIV-reactive and non-reactive PrEP participants. Given the variations in human behavior and HIV exposure, however, nonhuman primate models have great advantages for further study of the consequences of chemo-vaccination. Animals can be sampled very frequently; in contrast, in clinical trials, the larger intervals between visits make it more likely that chemo-vaccination effects are missed.

In conclusion, intermittent Truvada-based oral PrEP regimens can facilitate induction of T cell immunity following repeated virus exposure. Occurrence and implications of such chemo-vaccination effects should be evaluated in specimens from current human PrEP trials. We suggest that combination of PrEP and vaccines might be more efficacious than either intervention alone. Such studies can be informed by comprehensive examination of immune responses and outcomes of vaccines, PrEP, microbicides, and other combination approaches aimed at interrupting HIV transmission.

## Materials and Methods

### Ethics statement

The Institutional Animal Care and Use Committee (IACUC) of the Centers for Disease Control and Prevention (CDC) approved all macaque procedures described (protocol permit numbers: 1414OTTMONC, 1615GARMONC, 2099GARMONC). This study was carried out in strict accordance with the recommendations in the Guide for the Care and Use of Laboratory Animals of the National Institutes of Health (NIH) and with the recommendations of the Weatherall report, “The use of non-human primates in research”. All procedures were performed under anesthesia using ketamine, and all efforts were made to minimize suffering, improve housing conditions, and to provide enrichment opportunities (e.g., objects to manipulate in cage, varied food supplements, foraging and task-oriented feeding methods, interaction with caregivers and research staff).

### Macaques, virus, and repeat-low dose (RLD) virus challenges

Nine adult, male Indian rhesus macaques were housed at CDC. The genetic identity of eight MHC alleles with potential impact on SHIV susceptibility and infection course were (macaque ID number, identified alleles): 35451: B08; 33756: A01, A02; 4284: A08; 33246: A08; 35032: none of the above; 34912: A08, B17; AG94: A08; AI22: B01; 35720: A01, A08, B17 (evaluated at the University of Wisconsin AIDS Vaccine research laboratory). SHIV_SF162P3_ (SIVmac239 backbone plus HIV-1 B env) [32], [33] was provided by the NIH AIDS Research and Reference Reagent Program (NARRRP, catalog #6526). SHIV_SF162P3_ infection is typically accompanied by T and B cell responses [29], [34], thus allowing us to study formation of such responses during PrEP. All macaques underwent up to 14, once-weekly, rectal SHIV_SF162P3_ exposures at a low dose of 10 TCID_50_ per exposure as published elsewhere [16]. The dose equaled 7.6×10^5^ RNA copies, which is in the range of HIV-1 RNA amounts in human semen (10^3^–10^6^ viral copies per milliliter) during acute infection [35]. Virus exposures were stopped when a macaque became SHIV RNA-positive. Procedures for blood collection, and determination of SHIV infection with three methods (quantitation of viral RNA≥50 copies/mL plasma, serum anti-HIV antibody and proviral DNA determination) have been published [12], [16]. The HIV-1/HIV-2 plus O EIA (BioRad/Genetic Systems, Redmond, WA) antibody detection kit was utilized to determine time-point of seroconversion, using the cut-off criteria set forth by the manufacturer. This assay can detect both IgG and IgM, resulting in a higher sensitivity during early infection than commercial Western Blotting assays. It contains these antigens: recombinant HIV-1 gp160 (env) and p24, a Group O gp41 (env) peptide, and a gp36 (env) peptide from HIV-2. Since SHIV_SF162P3_ contains HIV-1-env, infected monkeys show HIV-1-env reactivity. In addition, there is a large amount of cross reactivity between antibodies to p27 from SIV/HIV-2 and HIV-1 p24. After a washout period of 28 weeks, four PrEP-protected animals were re-enrolled in an efficacy study that evaluated the impact of the M184V mutation associated with FTC resistance on PrEP efficacy. These 4 animals were exposed rectally to SHIV_SF162P3-M184V_ under the same conditions and using the same inoculation protocol. The dose of virus was increased to 40 TCID_50_ to account for a reduced transmissibility associated with M184V [14]. The M184V mutation is not expected to significantly affect overall anti-SHIV T cell responses, because it only alters one potential T cell epitope in *pol* gene products.

#### Intermittent PrEP regimen

Six macaques received human-equivalent doses of Truvada (22 mg/kg of TDF and 22 mg/kg of Emtricitabine FTC) by oral gavage seven days before viral challenge and 2 hours afterwards, and then once a week, 2 hours after each virus exposure. Thus, each virus exposure was flanked by a waning drug dose of 7 days prior, and one drug dose administered soon after exposure, a potential scenario for intermittent PrEP use in humans. Three additional macaques did not receive drug and were used as controls. The efficacy of the PrEP regimen and the kinetics of infection in breakthrough animals have been reported elsewhere [16].

#### T cell analyses by IFNγ-ELISPOTs

PBMC were enumerated with an automated Guava cell counter (Millipore, Billerica, MA). We enumerated SHIV-specific T cells by IFNγ-ELISPOTs, incubating fresh cells for 36 hours with the following peptide pools (15-mers with 11 amino acid overlaps) at a final concentration of 1.5 ug/ml: SIVmac239-pol (pol 1: peptides 1–50; pol 2: peptides 51–100; pol 3: peptides 101–150; pol 4: peptides 151–200; pol 5: peptides 201–263, NARRRP # 6443); HIV-1 consensus B-tat (NARRRP #5138), and SIVmac239-nef, vpr, or vif (NARRRP # 8762, 6449, 6205). Two SIVmac239-gag, 3 SHIV_SF162P3_-env pools, Staphylococcus aureus Enterotoxin (SEB, positive control) and a mock peptide pool (negative control) have been previously described [17]. Results were read on a S5 Core Analyzer (Cellular Technology Ltd, Shaker Heights, OH). The number of SHIV-specific T cells responding to each peptide pool was determined as SFU/10∧6 PBMC after subtracting the values for the mock peptide pool.

#### Statistical Methods

We established a strict cut-off value for IFNγ ELISPOTs to discriminate background variations from SHIV-specific T cells. This consisted of baseline T cell reactivity in the 9 virus-naïve macaques, cumulative of responses to all 14 peptide pools, plus 3 standard deviations (417 SFU/10^6^ PBMC). To analyze epitope specificity, the relative contribution of *gag* (pools 1+2), *env* (pools 1-3), *pol* (pools 1-5), *tat*, *nef*, *vpr*, or *vif* pools to the combined T cell response (all peptide pools) was calculated. Statistical analyses of epitope specificities were performed using unpaired, two-sided student's t-tests on results from groups of macaques (e.g. PrEP-treated uninfected macaques compared to infected macaques).

#### Flow cytometry

Prior to flow cytometry, PBMCs were frozen and thawed as described [17]. For intracellular cytokine analysis and effector and central memory T cell determination (T_EM_ and T_CM_) based on cytokine expression [18], we washed cells twice, and incubated them with their two dominant peptide pools (as determined by ELISPOT) at 1.5 ug/mL and Golgi-plug kit (BD Biosciences, San Jose, CA) for 6 hours. Antibody staining (clone names in parentheses, all from BD Biosciences, San Jose CA, unless otherwise stated) was performed with anti-CD3 (SP34-2), -CD4 (L200, NIH Nonhuman Primate Reagent Resource), -CD8 (SK1), -CD69 (L78), -CCR7 and -MIP-1β (unnamed, and D21-1351, R&D Systems Minneapolis, MN), -CD28 (CD28.2), -IL-2 (MQ1-17H12), -TNFα (MAB11), -IFNγ (B27). Samples were examined on a LSRII flow cytometer (BD Biosciences, San Jose, CA), and analyzed using FlowJo software 7.2.1 (Tree Star, San Carlos, CA). To analyze cytokine production quantitatively, we counted total lymphocytes/mL fresh blood, measured percentages of cytokine^+^ cells in thawed lymphocytes/mL of blood, and then calculated numbers of cytokine^+^ cells/mL blood. For quantitative T_EM_ and T_CM_ analysis, we gated on CD3^+^ and CD69^+^ cells. We then enumerated cytokine producing cells (making IFNγ, TNFα, MIP-1β, and IL-2 alone or simultaneously in any combination) as described above. T_CM_ cells were defined as CD3^+^ CD69^+^ (IFNγ, TNFα, MIP-1β, and/or IL-2)^+^, CD28^high^, CCR7^+^; while T_EM1_ cells were CD3^+^ CD69^+^ (IFNγ, TNFα, MIP-1β, and/or IL-2)^+^, CD28^high^, CCR7^−^; and T_EM2_ cells were CD3^+^ CD69^+^ (IFNγ, TNFα, MIP-1β, and/or IL-2)^+^, CD28^low^, CCR7^−^. Gates for T_EM_ and T_CM_ cells were set based on CD28 and CCR7 expression in control analyses of all CD3^+^ cells, regardless of their cytokine-production status.
